# High‐Rate, Large Capacity, and Long Life Dendrite‐Free Zn Metal Anode Enabled by Trifunctional Electrolyte Additive with a Wide Temperature Range

**DOI:** 10.1002/advs.202201433

**Published:** 2022-05-26

**Authors:** Chuyuan Lin, Xuhui Yang, Peixun Xiong, Hui Lin, Lingjun He, Qi Yao, Mingdeng Wei, Qingrong Qian, Qinghua Chen, Lingxing Zeng

**Affiliations:** ^1^ Engineering Research Center of Polymer Green Recycling of Ministry of Education College of Environmental Science and Engineering Fujian Normal University Fuzhou Fujian 350007 P. R. China; ^2^ School of Chemical Engineering Sungkyunkwan University Suwon‐si Gyeonggi‐do 16419 Republic of Korea; ^3^ Fujian Key Laboratory of Pollution Control & Resource Reuse Fuzhou Fujian 350007 P. R. China; ^4^ Fujian Provincial Key Laboratory of Electrochemical Energy Storage Materials Fuzhou University Fuzhou Fujian 350002 P. R. China; ^5^ Key Laboratory of Advanced Energy Materials Chemistry (Ministry of Education) College of Chemistry Nankai University Tianjin 300071 P. R. China

**Keywords:** electrolyte additive, homogenize Zn deposition, stable interface pH, Zn anode

## Abstract

Aqueous Zn‐ion batteries (AZIBs) have been recognized as promising energy storage devices due to their high theoretical energy density and cost‐effectiveness. However, side reactions and Zn dendrite generation during cycling limit their practical application. Herein, ammonium acetate (CH_3_COONH_4_) is selected as a trifunctional electrolyte additive to enhance the electrochemical performance of AZIBs. Research findings show that NH_4_^+^ (oxygen ligand) and CH_3_COO^–^ (hydrogenligand) with preferential adsorption on the Zn electrode surface can not only hinder Zn anode directly contact with active H_2_O, but also regulate the pH value of the electrolyte, thus suppressing the parasitic reactions. Additionally, the formed SEI is mainly consisted of Zn_5_(CO_3_)_2_(OH)_6_ with a high Zn^2+^ transference number, which could achieve a dendrite‐free Zn anode by homogenizing Zn deposition. Consequently, the Zn||Zn symmetric batteries with CH_3_COONH_4_‐based electrolyte can operate steadily at an ultrahigh current density of 40 mA cm^–2^ with a cumulative capacity of 6880 mAh cm^–2^, especially stable cycling at −10 °C. The assembled Zn||MnO_2_ full cell and Zn||activated carbon capacitor also deliver prominent electrochemical reversibility. This work provides unique understanding of designing multi‐functional electrolyte additive and promotes a long lifespan at ultrahigh current density for AZIBs.

## Graphical abstract

Zn metal anode enabled by CH_3_COONH_4_ electrolyte additive for dendrite‐free embraced extraordinary rate‐performance and high reversibility with a cumulative capacity of 6880 mAh cm^–2^ at an ultrahigh current density of 40 mA cm^–2^, superior to other Zn anode. The Zn||Zn symmetrical cells with attractive cycling performance stabilize over 900 h at a low temperature of −10 °C.

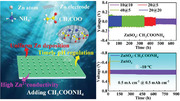

## 1 Introduction

Lithium‐ion batteries (LIBs) have taken the leading position as rechargeable and environmentally benign energy storage equipment according to the lack of memory effects and high energy densities.^[^ ^1^, ^2^, ^3^, ^4^ ^]^ Nevertheless, due to the limited resources and uneven distribution of lithium in the Earth's crust, it is highly desirble to exploite alternatives to LIBs.^[^ ^5^, ^6^, ^7^ ^]^ In the recent years, rechargeable aqueous zinc ion batteries (AZIBs) have lately drawn intensive attentions, mainly owing to their rich resources, cost‐effectiveness, environmental robustness, and high‐safety.^[^ ^8^, ^9^, ^10^, ^11^, ^12^ ^]^ Furthermore, the zinc metal possesses a higher theoretical capacity density (5855 mAh cm^–3^) and low redox potential (−0.763 V vs standard hydrogen electrode). Unfortunately, the problem of interfacial parasitic reactions, corrosion, and formation of zinc dendrite during the Zn plating/stripping process have hindered their commercialized application, which leads to poor utilization, lower Coulombic efficiency (CE), and short cycling lifespans of zinc metal anodes. Therefore, designing dendritic‐free and stable Zn anodes for AZIBs is a pressing task.^[^ ^13^, ^14^, ^15^, ^16^ ^]^

Several modification strategies have been proposed to build durable and high‐performance Zn anodes, including surface/interface adjustment and construction, electrode/host structure design, electrolyte optimization, and separator modification.^[^ ^17^, ^18^, ^19^, ^20^ ^]^ Among them, electrolyte optimization comprises high concentration electrolytes, quasi/solid‐state electrolytes, and electrolyte additives. Therefore, the electrolyte additives with the advantages of simplicity, high efficiency, and low‐cost are regarded as one of the most promising ways to stabilize Zn anodes.^[^ ^21^, ^22^, ^23^ ^]^ To date, a series of studies have been researched on the development of electrolyte additives. For instance, Wang et al. have proved that the decomposition of H_2_O was eliminated by the preferential solvation of dimethyl sulfoxide (DMSO) with Zn^2+^ and the strong interaction of H_2_O‐DMSO.^[^ ^24^ ^]^ In addition, Chen's group adopted a saccharin additive into the electrolyte to reduce the proportion of H_2_O in the electrical double layer through the characteristic adsorption of saccharin anions on the zinc anode, thus inhibiting the side reaction of the electrode‐electrolyte interface.^[^ ^25^ ^]^ Other electrolyte additives such as glucose,^[^ ^26^ ^]^ ethylenediaminetetraacetic acid tetrasodium salt,^[^ ^27^ ^]^ glycerol,^[^ ^28^ ^]^ 1‐ethyl‐3‐methylimidazolium chloride,^[^ ^15^ ^]^ ethyl ether,^[^ ^29^ ^]^ and lithium chloride,^[^ ^30^ ^]^ were also studied, and the progress of many efforts was obvious. However, attention is still limited to the pH of the electrolyte, which plays a crucial role in restricting side reactions and improving interface stability during cycling,^[^ ^31^, ^32^, ^33^ ^]^ especially at high current densities.

Recently, the demand for commercial energy storage applications operated in extreme weather or low‐temperature conditions has risen.^[^ ^34^, ^35^, ^36^ ^]^ For instance, AZIBs and Zn‐ion‐based capacitors that maintain stable performance under the subzero temperatures are urgently needed.^[^ ^37^, ^38^, ^39^ ^]^ The aqueous electrolytes will freeze at low temperature (<0 °C), which not only decreases the migration rate of Zn^2+^, but also causes a sharp fluctuation in pH at the interface within the Zn electrode/aqueous electrolyte. As a result, the overpotential is high, CE is poor, and the cycling life is limited. Therefore, it is vital to maintain the pH value of the electrolyte in a timely manner when operating at subzero temperatures, and less attention has been paid to these issues.

**Figure 1:**
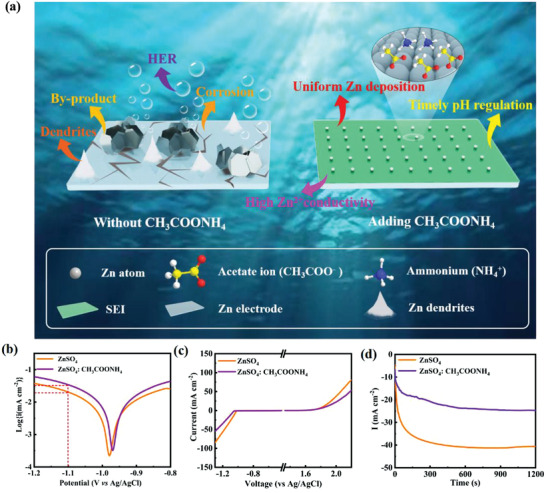
a) Schematic illustration of Zn surface evolution in electrolytes with/without CH_3_COONH_4_ additive. b) The Tafel plots were measured in ZnSO_4_: CH_3_COONH_4_ and ZnSO_4_ electrolyte at 1 mV s^−1^. c) Linear sweep voltammetry curves (LSV) were tested in ZnSO_4_: CH_3_COONH_4_ and ZnSO_4_ electrolyte. d) The chronoamperometry (CA) transient curves were accomplished in electrolytes with/without CH_3_COONH_4_ additive using a three‐electrode system.

Herein, a trifunctional electrolyte additive, ammonium acetate (CH_3_COONH_4_), is initially introduced in aqueous ZnSO_4_ electrolyte to restrain H_2_ evolution and maintain the pH value of the electrolyte (as shown in **Figure** **1**). Both characterization analysis and computational calculation results reveal that NH_4_^+^ and CH_3_COO^–^ exhibit outstanding zincophilicity, can preferentially adsorb on the Zn metal surface which effectively blocks the direct contact of H_2_O with the Zn anode and shields the tip effect to realize dendrite‐free Zn deposition.^[^ ^40^, ^41^, ^42^, ^43^ ^]^ More encouragingly, pH‐buffered CH_3_COONH_4_ can maintain the concentrations of OH^–^ and H^+^ at the electrode‐electrolyte interface, which achieves secondary protection for Zn anodes.^[^ ^32^ ^]^ Consequently, with the assistance of CH_3_COONH_4_ additive, the Zn||Zn symmetric cell sustains a long‐term cycle lifespan of over 2400 h at 2 mA cm^–2^, and high cycling stability at ultrahigh current densities (650 h at 10 mA cm^–2^ and 344 h at 40 mA cm^–2^). Moreover, the ZnSO_4_: CH_3_COONH_4_ electrolyte effectively improves the electrochemical performance of Zn‐based electrochemical energy storage devices based on MnO_2_ and activated carbon (AC) cathodes.

## 2 Results and Discussion

To systematically elucidate the effect of the CH_3_COONH_4_ additive on the side reaction of the Zn electrode, electrochemical tests were implemented. Specifically, the addition of CH_3_COONH_4_ resulted in the corrosion potential shifting positively (from 0.980 to 0.969 V) and the corrosion current decreasing by 0.252 mA cm^–2^ compared with that in the ZnSO_4_ electrolyte (Figure 1b), demonstrating that the corrosion tendency and the corrosion rate of the Zn anode were decreased.^[^ ^44^ ^]^ As shown in Figure 1c, the initial potential of the hydrogen evolution reaction (HER) with the ZnSO_4_: CH_3_COONH_4_ electrolyte was slightly reduced from −1.01 to −1.05 V, reflecting that the water‐induced HER was suppressed.^[^ ^45^ ^]^ Additionally, the current response increased significantly within 1200 s in the ZnSO_4_ electrolyte (Figure 1d), indicating an expansion in the effective electrode area.^[^ ^41^, ^46^ ^]^ Conversely, there was a comparatively slight increase of current density for the Zn anode in ZnSO_4_: CH_3_COONH_4_ electrolyte. This result revealed that denser deposition of the zinc surface can occur with the CH_3_COONH_4_ additive.

**Figure 2:**
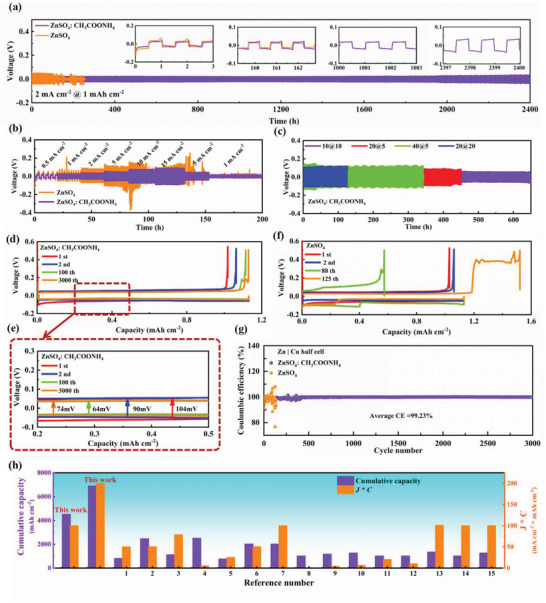
a) Long‐term performance of Zn||Zn symmetrical batteries with different electrolytes at 2 mA cm^–2^ for 1 mAh cm^–2^. b) Rate performance of symmetrical cells with different electrolytes. c) Cycling performance of Zn||Zn symmetrical cells with ZnSO_4_: CH_3_COONH_4_ at 10@10, 20@5, 40@5, and 20 mA cm^–2^@20 mAh cm^–2^. The plating/striping curves of Zn||Cu cells d, e) with CH_3_COONH_4_ additive; f) without CH_3_COONH_4_ additive; g) the corresponding CE. h) Comparison of cumulative capacity and *J × C* in symmetrical cells with recent reports.

The promotional function of the ZnSO_4_: CH_3_COONH_4_ electrolyte on the electrochemical stability of the Zn electrode was examined by assembling Zn||Zn symmetric cells into the long‐term galvanostatic cycle. **Figure** **2**a depicts that the symmetric cell with the ZnSO_4_ electrolyte experienced the short circuit after 200 h at 2 mA cm^−2^ for 1 mAh cm^−2^, which could be attributed to HER and dendrite growth. In stark contrast, at 2 and 4 mA cm^−2^ (Figure S1, Supporting Information) for 1 mAh cm^−2^, the zinc symmetric cells in the ZnSO_4_: CH_3_COONH_4_ electrolyte cycled for 2400 and 1600 h with steady overpotentials of 29 and 59 mV, respectively, illustrating that the Zn anode with CH_3_COONH_4_ additives exhibited desirable cycling stability under different current densities.

The results of the rate performance tests showed that at the same area capacity of 1 mAh cm^–2^, the Zn||Zn symmetrical batteries enabled to be consistently stable for 20 h in the ZnSO_4_: CH_3_COONH_4_ electrolyte, even if the current density was up to 15 mA cm^–2^. However, the cell with ZnSO_4_ electrolyte was almost inoperative at the test condition of 5 mA cm^–2^ because of severe polarization (Figure 2b). It was discovered that the cycling time of the Zn||Zn batteries with ZnSO_4_: CH_3_COONH_4_ electrolyte reached 650, 450, and 120 h at 10@10, 20@5 and 20 mA cm^–2^@20 mAh cm^–2^, respectively. More notably, the cells with the modified electrolyte could operate steadily for 344 h at a super‐high current density of 40 mA cm^–2^ and maintain the overpotential in the acceptable range (≈90 mV), which is superior to most of the previous reports (Figure 2c). In addition, the average CE of the Zn||Cu cell with the ZnSO_4_: CH_3_COONH_4_ electrolyte was up to 99.23% even after 3000 cycles (Figure 2d–g), revealing the fascinating reversibility of Zn plating/stripping. Compared to recent reports,^[^ ^26^, ^42^, ^44^, ^45^, ^47^, ^48^, ^49^, ^50^, ^52^, ^53^, ^54^, ^55^, ^56^, ^57^, ^58^ ^]^ the cumulative capacity of the zinc anode was as high as 6880 mAh cm^–2^ with the modification of CH_3_COONH_4_ additive, and the product of the maximum current density and areal capacity could reach to 200 (Figure 2h), proving that our work fulfills the requirements of high capacity, large current, and long cycle life, which is hopeful for application in practical production.

**Figure 3:**
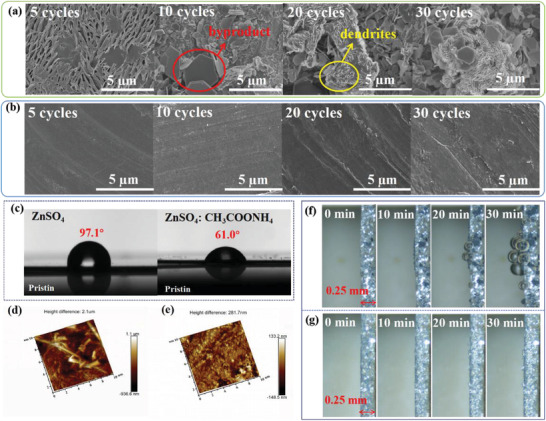
SEM images of Zn||Zn symmetrical cells: a) ZnSO_4_ electrolyte; b) ZnSO_4_: CH_3_COONH_4_ electrolyte. c) The contact angle of different electrolytes on Zn foil. d) AFM images of Zn surface after cycling in ZnSO_4_ electrolyte. e) AFM images of Zn surface after cycling in ZnSO_4_: CH_3_COONH_4_ electrolyte. In situ optical microscope image of Zn electrode after cycling for 0, 20, 40, and 30 min: f) ZnSO_4_ electrolyte; g) ZnSO_4_: CH_3_COONH_4_ electrolyte.

Scanning electron microscopy (SEM) characterization was utilized to probe the inhibitory effect of the additives on dendrite growth, as presented in **Figure** **3**a,b. The surface of the Zn anode becomes rough after just 5 cycles in ZnSO_4_ electrolyte, which becomes severely aggravated with the presence of hexagonal nanosheets and Zn dendrites after 30 cycles (Figure 3a). In contrast, the surface morphology of the zinc plate remains planar after cycling 30 times in the ZnSO_4_: CH_3_COONH_4_ electrolyte at 5 mA cm^–2^ (Figure 3b). The morphology of the Zn electrode cycled for 200 h further verified the uniform and planar distribution of Zn on the contact face of the Zn electrode under the action of CH_3_COONH_4_, which is consistent with the above results of the CA test (Figure S2, Supporting Information). As depicted in Figure 3c and Figure S3, Supporting Information, the smaller contact angle between the modified electrolyte and Zn plate before and after cycling (61.0 and 52.5°) apparently illuminates the enhancement of hydrophilicity between the electrode and the optimized electrolyte. Theoretically, the strong hydrophilicity could be beneficial for reducing the interfacial free energy and thus facilitate the average deposition of Zn.^[^ ^21^, ^31^, ^59^, ^60^ ^]^ Moreover, the ion conductivity of the electrolyte was elevated in the presence of CH_3_COONH_4_ (Figure S4, Supporting Information)_._ As expected, the results of the atomic force microscope (AFM) tests further confirmed that there was a significant reduction in the roughness of the cycled Zn anode surface in the presence of additives (Figure 3d–e). Optical in situ microscopy was applied to visually observe the reaction of the Zn anode‐electrolyte interface during cycling at 10 mA cm^–2^ (Figure 3f,g and Video S1, Supporting Information). Not surprisingly, Zn dendrites and H_2_ bubbles appeared simultaneously at the surface of the zinc metal anode after cycling in the ZnSO_4_ bare electrolyte within 30 min. Conversely, almost no Zn dendrites and bubbles were observed for the zinc electrode in the CH_3_COONH_4_‐containing electrolyte. In addition, the photos in Figure S5, Supporting Information, shows that the electrode shell of the Zn||Zn cell with ZnSO_4_ electrolyte swelled up owing to gas generation after cycling for 50 times at 20 mA cm^–2^, while the cell using ZnSO_4_: CH_3_COONH_4_ electrolyte sealed well. Accordingly, there are reasons to believe that CH_3_COONH_4_ can mitigate dendrite growth and reduce corrosion, which enables the enhancement of the invertibility of the zinc electrode.

**Figure 4:**
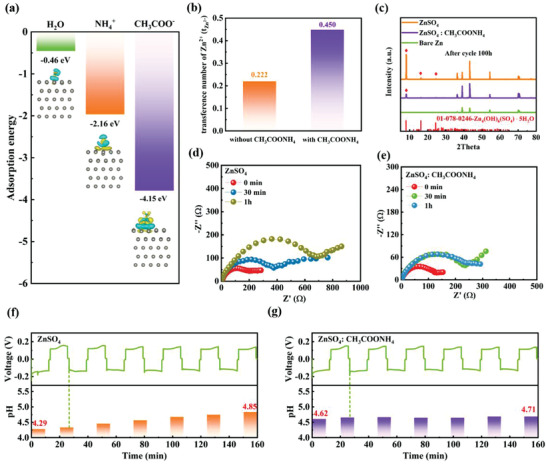
a) The adsorption energy of H_2_O, NH_4_^+^, and CH_3_COO^–^ on Zn (0001) surface. b) The Zn‐ion transference number (*t*_Zn_^2+^) of a bare Zn electrode and a SEI‐Zn electrode. c) X‐ray diffraction (XRD) patterns of pristine Zn foil and Zn‐electrode after cycling for 100h in electrolytes with/without CH_3_COONH_4_ additive. d, e) Electrochemical impedance spectroscopy (EIS) diagram of the symmetric batteries (cycling 5 times at the current density of 1 mA cm^−2^) in ZnSO_4_ electrolytes and ZnSO_4_: CH_3_COONH_4_ electrolytes before and after resting for 30 min and 1 h, respectively. f, g) pH monitoring of ZnSO_4_ and ZnSO_4_: CH_3_COONH_4_ electrolyte of symmetric batteries cycling at 5 mA cm^−2^, respectively.

In addition, the solid electrolyte interphases (SEI) formation of Zn anodes in the modified electrolyte was also explored. As depicted in Figure S6, Supporting Information, the composition of the surface of Zn anodes cycled in different electrolytes was characterized by X‐ray photoelectron spectroscopy (XPS). Notably, Zn anodes cycled in ZnSO_4_: CH_3_COONH_4_ electrolytes exhibited more pronounced C 1s (284.8, 286.1, and 289.5 eV) and N 1s signals (401.2, 399.8, and 398.8 eV) belonging to CH_3_COO^–^ and NH_4_^+^, respectively, partially adsorbed on the surface of Zn foil. Nevertheless, the N 1s spectrum of the zinc anode in the ZnSO_4_ electrolyte surface shows no significant diffraction peaks, and the peak density of C═O is extremely weak. To corroborate the mechanism of the protective barrier on the Zn anode surface, the absorption energies of H_2_O, NH_4_^+^, and CH_3_COO^–^ on Zn (0001) were calculated through density functional theory (DFT). **Figure** **4**a shows that the adsorption energies of NH_4_^+^ and CH_3_COO^–^ on Zn (0001) are much lower than that of H_2_O, demonstrating that NH_4_^+^ and CH_3_COO^–^ are adsorbed on the Zn metal surface to form an ion protective layer during cycling, which effectively blocks the direct contact of water molecules with the zinc electrode.

To investigate the detailed component of the SEI layer, scanning electron microscope ‐ energy dispersive spectroscopy (SEM‐EDS) characterization for the cycled Zn anode in ZnSO_4_: CH_3_COONH_4_ electrolyte was carried out, it was found that the SEI layer with a thickness of about 2 µm was formed on the anode surface after cycling (Figure S7, Supporting Information). And the result of in‐depth XPS facilitated by Ar^+^ sputtering in Figure S8, Supporting Information, shows that CO_3_^2–^ appeared at 290.2 eV in the C 1s spectrum by sputtering exceeded 60 s, and the O–C═O bond originated from CH_3_COO^–^ gradually disappears. While the N species vanished from the zinc anode surface after Ar^+^ sputtering. With the extension of etching time, the dominant signal of the Zn spectrum distinctly shifts from Zn–O (≈1022.4 eV) to Zn metal (≈1021.7 eV), further verifying that the formation of the protective SEI layer on the Zn anode, which is consistent with the characterization results of SEM‐EDS. In Figure S10, Supporting Information, the electrochemical impedance sepctroscopy of the Zn||Zn cells proved the SEI formation of Zn anodes in the modified electrolyte. Furthermore, X‐ray diffraction (XRD) patterns further demonstrated that the Zn_5_(CO_3_)_2_(OH)_6_ component was produced on the surface of Zn anode after cycling in ZnSO_4_: CH_3_COONH_4_ electrolyte (Figure S9, Supporting Information). However, it seems impossible that the CO_3_^2–^ species was derived from CH_3_COO^–^ by oxidizing on the Zn anode surface for the exorbitant energy barrier. Accordingly, it is more reasonable to conclude that the formation of CO_3_^2–^ is attributed to the dissolved CO_2_ from the ambient environment (Equation 1).^[^ ^51^ ^]^ As displayed in Figure 4g, the increase of pH value of the electrolyte with CH_3_COONH_4_ facilitated CO_2_ to dissolve in the solution. Therefore, CO_3_^2–^ and OH^–^ (stemmed from HER, Equation 2) combine with Zn^2+^ to produce Zn_5_(CO_3_)_2_(OH)_6_ (Equation 3), which is the main component of the formed SEI with lower solubility and high ion conduction. The formation mechanism of Zn_5_(CO_3_)_2_(OH)_6_:

(1) $$CO_{2}+H_{2}O⇌{\mathrm{HCO}}_{3}^{-}+H^{+}⇌{\mathrm{CO}}_{3}^{2-}+2H^{+}$$

(2) $$H_{2}O⇌OH^{-}+H^{+}$$

(3) $$2{\mathrm{CO}}_{3}^{2-}+6{\mathrm{OH}}^{-}+5{\mathrm{Zn}}^{2+}\to {\mathrm{Zn}}_{5}{\left({\mathrm{CO}}_{3}\right)}_{2}{\left(\mathrm{OH}\right)}_{6}$$

The Zn‐ion transference number (*t*_Zn_^2+^) of the SEI formed in the modified electrolyte was larger than that in the ZnSO_4_ electrolyte (Figure 4b), which means the smaller concentration gradient of Zn^2+^ on the electrode surface and the more uniform field strength distribution, it is instrumental in homogenizing the deposition of Zn^2+^ and is less likely to generate harmful dendrites.^[^ ^52^, ^61^ ^]^ The detailed calculation process of the *t*_Zn_^2+^ results is shown in Figure S11, Supporting Information. Meanwhile, conformed to the Arrhenius equation, the activation energy (*E*a) of the Zn^2+^ de‐solvation in different interfaces was obtained by fitting the corresponding Nyquist plots of Zn symmetric cells with different kinds of electrolyte from 298.15 to 323.15 K (Figures S12 and S13, Supporting Information). Note that the activation energy of Zn^2+^ deposition within the electrode/electrolyte interface reduced from 42.4 to 35.4 kJ mol^–1^ with the assistance of CH_3_COONH_4_, which indicated that the more rapid diffusion of Zn^2+^ to homogenize the interfacial Zn^2+^ flux, thus leading to uniform Zn deposition on the anode surface. Moreover, the nucleation overpotential (NOP) in ZnSO_4_: CH_3_COONH_4_ electrolyte increased by 14.2 mV at the first cycle compared with ZnSO_4_ electrolyte (Figures S14 and S15, Supporting Information), which principally resulted from the coordination between Zn^2+^ ions and CH_3_COO^–^. Nevertheless, the NOP in ZnSO_4_: CH_3_COONH_4_ electrolyte decreased from 58.7 to 37.0 mV after cycling for three times due to the interphase with high Zn^2+^ ion transference number, which reduced the energy barriers of interfacial ion transport to realize kinetically fast and dendrite‐free zinc deposition. Combined with the SEM image of Ti foil after cycling in Figure S16, Supporting Information, it was further proved that the ion layer and the formed SEI pregnant with Zn_5_(CO_3_)_2_(OH)_6_ plays a significant role in the inhibition of dendrite formation.

Actually, the phenomenon of corrosion of Zn metal in mildly acidic ZnSO_4_ is especially pronounced (Figures S17 and S18, Supporting Information). The XRD pattern (Figure 4c) further showed that after 100 h of cycling in the ZnSO_4_ electrolyte, a large amount of Zn_4_SO_4_(OH)_6_·5H_2_O was generated on the interface of the zinc anode. In sharp contrast, the diffraction peak intensity of Zn_4_SO_4_(OH)_6_·5H_2_O on the contact face of the zinc electrode after cycling in the modified electrolyte can almost negligibly, suggesting that the SEI formed can evidently ameliorate the thermodynamic steadiness of Zn, which could be propitious to enhance the long‐term cycle performance of AZIBs,^[^ ^38^ ^]^ particularly at ultrahigh current density (40 mAh cm^–2^). Compared to the use of ZnSO_4_ bare electrolyte, the cells in the electrolyte with additives exhibited a stable resistance of ≈250 Ω with the extension of standing time. The phenomenon further demonstrated that the SEI protective layer could effectively alleviate the adverse chemical side reaction of interface within the Zn anode and the ZnSO_4_: CH_3_COONH_4_ electrolyte, to ensure excellent interface stability (Figure 4d–e and Figure S19, Supporting Information).^[^ ^53^, ^59^ ^]^

At the same time, the corrosion rate of the Zn anode under different electrolytes was evaluated by monitoring the pH during the symmetric cell cycle in real‐time (Figure S20, Supporting Information). As shown in Figure 4f‐g, the addition of ammonium acetate increased the pH of the electrolyte to 4.62 compared to the ZnSO_4_ electrolyte (pH = 4.29), mainly because NH_4_^+^ and CH_3_COO^–^ could balance the concentration of OH^–^ and H^+^ in the electrolyte. HER inevitably occurs during charging and discharging, which leads to corrosion of the Zn anode and a local rise in the pH value.^[^ ^62^, ^63^ ^]^ As observed, the pH value of the electrolyte without CH_3_COONH_4_ additive increased from 4.29 to 4.85 after 150 min of cycling at a current density of 5 mA cm^–2^, while the pH of the electrolyte with the CHCOONH_4_ held steady, implying that the pH buffered CH_3_COONH_4_ can remain the pH value of the electrolyte by combining CHCOO^–^ and NH_4_^+^ with the H^+^ and OH^–^ in the electrolyte respectively, which is capable of achieving secondary protection for the zinc electrode to suppress the electrochemical side reactions during the charge and discharge process. The pH evolution of the two electrolytes after cycled at a higher current density of 20 mA cm^–2^ was also monitored (Figure S21, Supporting Information). After 160 min, the pH of ZnSO_4_: CH_3_COONH_4_ electrolyte has a little increase from 4.65 to 4.83, and larger fluctuant from 4.12 to 4.80 was observed in ZnSO_4_ electrolyte, suggesting the CH_3_COONH_4_ additive still exhibits a great buffer function even at a higher current density. Moreover, pH evolution of the ZnSO_4_: CH_3_COONH_4_ electrolyte cycled at −10 °C was also verified. As shown in Figures S22 and S23, Supporting Information, there was no significant increase on the pH of the optimized electrolyte in the presence of CH_3_COONH_4_, and the cycle‐life of the corresponding zinc symmetric batteries was extended by more than 20 times at −10 °C. Based on a satisfactory pH buffering performance at low temperature, we concluded that the ZnSO_4_: CH_3_COONH_4_ electrolyte is provided with certain low‐temperature resistance.

**Figure 5:**
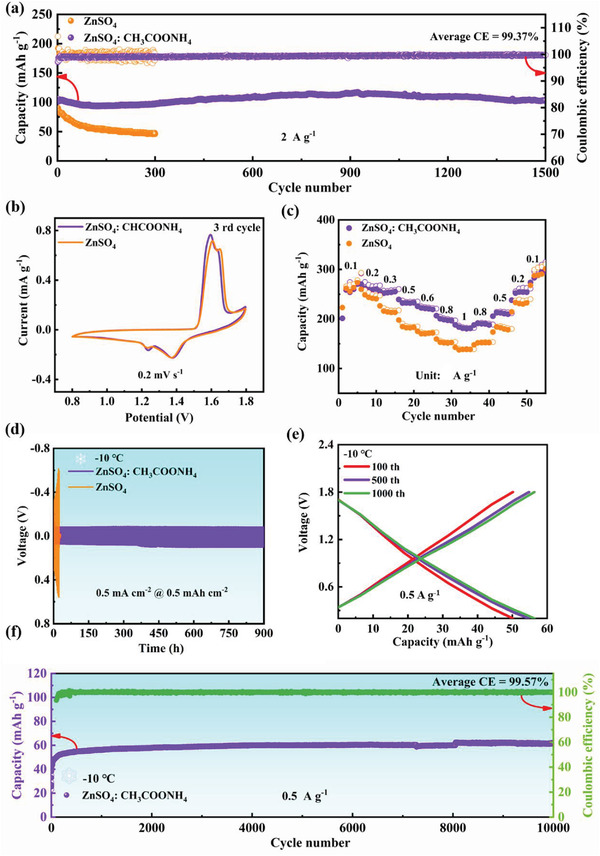
a) The long‐life cycle performance of Zn||MnO_2_ full batteries in ZnSO_4_: CH_3_COONH_4_ electrolyte and ZnSO_4_ electrolyte. b) CV curves of Zn||MnO_2_ batteries. c) Rate performance of Zn||MnO_2_ batteries in ZnSO_4_: CH_3_COONH_4_ electrolyte and ZnSO_4_ electrolyte. d) The long‐life cycle performance of zinc symmetrical cells in ZnSO_4_: CH_3_COONH_4_ electrolyte and ZnSO_4_ electrolyte at −10 °C. e) Charge and discharge curves of Zn||Ac capacitors at −10 °C. f) The cycle performance of Zn||Ac capacitors in ZnSO_4_: CH_3_COONH_4_ electrolyte at −10 °C.

To clarify the efficiency of the ZnSO_4_:CH_3_COONH_4_ electrolyte, we assembled a full cell using MnO_2_/carbon as the cathode, and SEM of MnO_2_/carbon is shown in Figure S24, Supporting Information. From the cyclic voltammetry (CV) curves in **Figure** **5**b and Figure S25, Supporting Information, the voltage windows of the full cells assembled with different electrolytes are identical. Furthermore, galvanostatic test results show that the full cell with the modified electrolyte could cycle at a high current density of 2 A g^–1^ for 1500 cycles with a specific capacity of ≈100 mAh g^–1^ and an average CE of 99.37% (Figure 5a). In contrast, the capacity retention of the full cell is already below 50% after 300 cycles with ZnSO_4_ electrolyte, which is attributed to side reactions such as hydrogen precipitation and dendrite growth.^[^ ^64^ ^]^ Distinctly, uniform and dense Zn deposition was obtained in ZnSO_4_: CH_3_COONH_4_ electrolyte, resulting in a flat surface of Zn anode (Figure S26, Supporting Information). Besides, the MnO_2_ cathode perfectly retains its pristine morphology after 100 cycles (Figure S26f, Supporting Information), which suggested that the CH_3_COONH_4_ additives can effectively suppress the corrosion and dendrite for the full cells. To further evaluate the application of electrolytes in different devices, Zn||AC capacitors were assembled using commercially available activated carbon as the positive electrode. The capacitors achieved more than 18000 cycles at a current density of 1 A g^–1^ at 25 °C, the average CE was more than 99.5% (Figures S27 and S28, Supporting Information). More surprisingly, the Zn||Zn symmetric cell with ZnSO_4_: CH_3_COONH_4_ electrolyte cycled for 900 h at −10 °C (Figure 5d). It can be seen intuitively in Figure S29, Supporting Information, that the modified electrolyte preserves the original liquid state at −10 °C. Additionally, the capacitor with ZnSO_4_: CH_3_COONH_4_ as the electrolyte was found to be stable for 10 000 cycles at this low temperature (Figure 5e–f), which indicates that the CH_3_COONH_4_ additive can not only prolong the cycle life of zinc ion batteries and zinc ion capacitors but also slightly improve the low‐temperature performance of AZIBs.

## 3 Conclusion 

In summary, we have discovered that by utilizing CH_3_COONH_4_ as an electrolyte additive, NH_4_^+^ and CH_3_COO^–^ with outstanding zincophilicity, enable adsorption on the Zn metal anode surface preferentially, which enables homogenization of zinc deposition and inhibites the formation of byproduct. Meanwhile, CH_3_COONH_4_ restrains the increase in local pH, which achieves secondary protection for the Zn anode. Based on the above mechanics, the test results further confirm that the introduction of CH_3_COONH_4_ successfully achieved a highly reversible Zn electrode in weakly acidic aqueous electrolytes. Specifically, zinc symmetric cells stably cycled over 2400 h at 2 mA cm^–2^ with a low overpotential of 29 mV, and over 600 h at a high‐current density of 10 mA cm^–2^ and a large‐capacity of 10 mAh cm^–2^. Furthermore, the Zn||Cu battery sustains over 3000 cycles with an average CE of 99.23%. In addition, the electrochemical reversibility of the Zn||MnO_2_ full cell was evidently improved by using a modified electrolyte (1500 cycles at 2 A g^–1^). More surprisingly, Zn||Zn symmetric cell work steadily for 900 h under harsh conditions at −10 °C, the Zn||AC capacitor with excellent cycling performance stabilizes over 10000 cycles at low temperature of −10 °C. Finally, this novel electrolyte additive provides a reference for the development of AZIBs with a wide temperature range in the future.

## 4 Experimental Section

### Preparation of Electrolytes and Electrodes

The ZnSO_4_ electrolytes were prepared by dissolving ZnSO_4_·7H_2_O in deionized water at room temperature and the ZnSO_4_: CH_3_COONH_4_ electrolytes were prepared by adding 20 mM ammonium acetate (CH_3_COONH_4_) into the 2 M ZnSO_4_ electrolytes. The MnO_2_/carbon cloth cathode was prepared by the electrodeposition method according to the previous research.^[^ ^37^ ^]^ Typically, the electrodeposition was conducted in a solution consisting of 0.1 M Mn(CH_3_COO)_2_·4H_2_O and 0.1 M Na_2_SO_4_ at 1.0 V for 300 s. More details of the material characterization and computational methods were provided in the Supporting Information.

### Electrochemical Measurements

The symmetric cells were assembled by using two pieces of Zn foils as two electrodes in CR2032‐type cell. Two different electrolytes (ZnSO_4_ and ZnSO_4_: CH_3_COONH_4_) each with 50 µL were dropped into the coin cell with a piece of glass fiber as a separator. The full cells were fabricated by using Zn plate, MnO_2_ cathode, and two different electrolytes (ZnSO_4_ with 0.2 M MnSO_4_ or ZnSO_4_: CH_3_COONH_4_ with 0.2 M MnSO_4_) and glass fiber. The galvanostatic cycling tests was tested in the voltage range of 0.8–1.8 V (vs Zn^2+^/Zn).

## Conflict of Interest

The authors declare no conflict of interest.

## Supporting information

Supporting Information

Supplemental Video 1

## Data Availability

The data that support the findings of this study are available from the corresponding author upon reasonable request.

## References

[1] D. Lin , Y. Liu , Y. Cui , Nat. Nanotechnol. 2017, 12, 194. 28265117 10.1038/nnano.2017.16

[2] B. Dunn , H. Kamath , J.‐M. J. S. Tarascon , Science 2011, 334, 928. 22096188 10.1126/science.1212741

[3] K. Turcheniuk , D. Bondarev , V. Singhal , G. Yushin , Nature 2018, 559, 467. 30046087 10.1038/d41586-018-05752-3

[4] J. Li , S. Zhang , Y. Wu , B. Jin , M. Shao , Mater. Today Energy 2021, 22, 100849.

[5] P. Chen , X. Yuan , Y. Xia , Y. Zhang , L. Fu , L. Liu , N. Yu , Q. Huang , B. Wang , X. Hu , Y. Wu , T. van Ree , Adv. Sci. 2021, 8, 2100309. 10.1002/advs.202100309 PMC8188195 34105273

[6] E. Fan , L. Li , Z. Wang , J. Lin , Y. Huang , Y. Yao , R. Chen , F. Wu , Chem. Rev. 2020, 120, 7020. 31990183 10.1021/acs.chemrev.9b00535

[7] F. Wan , X. Zhou , Y. Lu , Z. Niu , J. Chen , ACS Energy Lett. 2020, 5, 3569.

[8] F. Wang , O. Borodin , T. Gao , X. Fan , W. Sun , F. Han , A. Faraone , J. A. Dura , K. Xu , C. Wang , Nat. Mater. 2018, 17, 543. 29662160 10.1038/s41563-018-0063-z

[9] C. Zhang , J. Holoubek , X. Wu , A. Daniyar , L. Zhu , C. Chen , D. P. Leonard , I. A. Rodriguez‐Perez , J. X. Jiang , C. Fang , X. Ji , Chem. Commun. 2018, 54, 14097. 10.1039/c8cc07730d 30488907

[10] L. Yan , Y. Zhang , Z. Ni , Y. Zhang , J. Xu , T. Kong , J. Huang , W. Li , J. Ma , Y. Wang , J. Am. Chem. Soc. 2021, 143, 15369. 34491047 10.1021/jacs.1c06936

[11] Q. Zhang , J. Luan , Y. Tang , X. Ji , H. Wang , Angew. Chem., Int. Ed. 2020, 59, 13180. 10.1002/anie.202000162 32124537

[12] B. Deka Boruah , A. Mathieson , S. K. Park , X. Zhang , B. Wen , L. Tan , A. Boies , M. De Volder , Adv. Energy Mater. 2021, 11, 2100115.

[13] L. E. Blanc , D. Kundu , L. F. Nazar , Joule 2020, 4, 771.

[14] L. Ma , M. A. Schroeder , T. P. Pollard , O. Borodin , M. S. Ding , R. Sun , L. Cao , J. Ho , D. R. Baker , C. Wang , K. Xu , Energy Environ. Mater. 2020, 3, 516.

[15] Q. Zhang , Y. Ma , Y. Lu , X. Zhou , L. Lin , L. Li , Z. Yan , Q. Zhao , K. Zhang , J. Chen , Angew. Chem., Int. Ed. 2021, 60, 23357. 10.1002/anie.202109682 34382322

[16] B. Sun , Q. Zhang , W. Xu , R. Zhao , H. Zhu , W. Lv , X. Li , N. J. N. E. Yang , Nano Energy 2022, 94, 106937.

[17] L. Hong , X. Wu , C. Ma , W. Huang , Y. Zhou , K.‐X. Wang , J.‐S. Chen , J. Mater. Chem. A 2021, 9, 16814.

[18] L. Yuan , J. Hao , C.‐C. Kao , C. Wu , H.‐K. Liu , S.‐X. Dou , S.‐Z. Qiao , Energy Environ. Sci. 2021, 14, 5669.

[19] L. Hu , P. Xiao , L. Xue , H. Li , T. Zhai , Energy Chem. 2021, 3, 100052.

[20] W. Kao‐ian , M. T. Nguyen , T. Yonezawa , R. Pornprasertsuk , J. Qin , S. Siwamogsatham , S. Kheawhom , Mater. Today Energy 2021, 21, 100738.

[21] J. Hao , J. Long , B. Li , X. Li , S. Zhang , F. Yang , X. Zeng , Z. Yang , W. K. Pang , Z. Guo , Adv. Funct. Mater. 2019, 29, 1903605.

[22] D. Chao , S.‐Z. Qiao , Joule 2020, 4, 1846.

[23] P. Wang , X. Xie , Z. Xing , X. Chen , G. Fang , B. Lu , J. Zhou , S. Liang , H. Fan , Adv. Energy Mater. 2021, 11, 2101158.

[24] L. Cao , D. Li , E. Hu , J. Xu , T. Deng , L. Ma , Y. Wang , X. Q. Yang , C. Wang , J. Am. Chem. Soc. 2020, 142, 21404. 33290658 10.1021/jacs.0c09794

[25] C. Huang , X. Zhao , S. Liu , Y. Hao , Q. Tang , A. Hu , Z. Liu , X. Chen , Adv. Mater. 2021, 33, 2100445. 10.1002/adma.202100445 34338350

[26] P. Sun , L. Ma , W. Zhou , M. Qiu , Z. Wang , D. Chao , W. Mai , Angew. Chem., Int. Ed. 2021, 60, 18247. 10.1002/anie.202105756 34036748

[27] S. J. Zhang , J. Hao , D. Luo , P. F. Zhang , B. Zhang , K. Davey , Z. Lin , S. Z. Qiao , Adv. Energy Mater. 2021, 11, 2102010.

[28] Y. Zhang , M. Zhu , K. Wu , F. Yu , G. Wang , G. Xu , M. Wu , H.‐K. Liu , S.‐X. Dou , C. Wu , J. Mater. Chem. A 2021, 9, 4253.

[29] W. Xu , K. Zhao , W. Huo , Y. Wang , G. Yao , X. Gu , H. Cheng , L. Mai , C. Hu , X. Wang , Nano Energy 2019, 62, 275.

[30] X. Guo , Z. Zhang , J. Li , N. Luo , G.‐L. Chai , T. S. Miller , F. Lai , P. Shearing , D. J. L. Brett , D. Han , Z. Weng , G. He , I. P. Parkin , ACS Energy Lett. 2021, 6, 395.

[31] X. Zeng , J. Mao , J. Hao , J. Liu , S. Liu , Z. Wang , Y. Wang , S. Zhang , T. Zheng , J. Liu , P. Rao , Z. Guo , Adv. Mater. 2021, 33, 2007416. 10.1002/adma.202007416 33576130

[32] Q. Yang , L. Li , T. Hussain , D. Wang , L. Hui , Y. Guo , G. Liang , X. Li , Z. Chen , Z. Huang , Y. Li , Y. Xue , Z. Zuo , J. Qiu , Y. Li , C. Zhi , Angew. Chem., Int. Ed. 2022, 61, 202112304. 10.1002/anie.202112304 34799952

[33] D. Han , Z. Wang , H. Lu , H. Li , C. Cui , Z. Zhang , R. Sun , C. Geng , Q. Liang , X. Guo , Y. Mo , X. Zhi , F. Kang , Z. Weng , Q. H. Yang , Adv. Energy Mater. 2022, 10, 2102982.

[34] Q. Zhang , K. Xia , Y. Ma , Y. Lu , L. Li , J. Liang , S. Chou , J. Chen , ACS Energy Lett. 2021, 6, 2704.

[35] J. Zhao , J. Zhang , W. Yang , B. Chen , Z. Zhao , H. Qiu , S. Dong , X. Zhou , G. Cui , L. Chen , Nano Energy 2019, 57, 625.

[36] X. Zhu , C. Ji , Q. Meng , H. Mi , Q. Yang , Z. Li , N. Yang , J. Qiu , Small 2022, 18, 2200055. 10.1002/smll.202200055 35274442

[37] N. Chang , T. Li , R. Li , S. Wang , Y. Yin , H. Zhang , X. Li , Energy Environ. Sci. 2020, 13, 3527.

[38] J. Wu , Q. Liang , X. Yu , Q. F. Lü , L. Ma , X. Qin , G. Chen , B. Li , Adv. Funct. Mater. 2021, 31, 2011102.

[39] Z. Jian , N. Yang , M. Vogel , S. Leith , A. Schulte , H. Schönherr , T. Jiao , W. Zhang , J. Müller , B. Butz , X. Jiang , Adv. Energy Mater. 2020, 10, 2002202.

[40] L. Cao , D. Li , T. Deng , Q. Li , C. Wang , Angew. Chem., Int. Ed. 2020, 59, 19292. 10.1002/anie.202008634 32638488

[41] H. Yang , Y. Qiao , Z. Chang , H. Deng , X. Zhu , R. Zhu , Z. Xiong , P. He , H. Zhou , Adv. Mater. 2021, 33, 2102415. 10.1002/adma.202102415 34338385

[42] X. Xie , S. Liang , J. Gao , S. Guo , J. Guo , C. Wang , G. Xu , X. Wu , G. Chen , J. Zhou , Energy Environ. Sci. 2020, 13, 503.

[43] Y. Zeng , X. Zhang , Y. Meng , M. Yu , J. Yi , Y. Wu , X. Lu , Y. Tong , Adv. Mater. 2017, 29, 1700274. 10.1002/adma.201700274 28452147

[44] X. Zeng , K. Xie , S. Liu , S. Zhang , J. Hao , J. Liu , W. K. Pang , J. Liu , P. Rao , Q. Wang , J. Mao , Z. Guo , Energy Environ. Sci. 2021, 14, 5947.

[45] Q. Zhang , J. Luan , L. Fu , S. Wu , Y. Tang , X. Ji , H. Wang , Angew. Chem., Int. Ed. 2019, 58, 15841. 10.1002/anie.201907830 31437348

[46] P. Xiong , Y. Kang , H. Yuan , Q. Liu , S. H. Baek , J. M. Park , Q. Dou , X. Han , W.‐S. Jang , S. J. Kwon , Y.‐M. Kim , W. Li , H. S. Park , Appl. Phys. Rev. 2022, 9, 011401.

[47] L. Zhang , B. Zhang , T. Zhang , T. Li , T. Shi , W. Li , T. Shen , X. Huang , J. Xu , X. Zhang , Z. Wang , Y. Hou , Adv. Funct. Mater. 2021, 31, 2001867.

[48] T. Chen , Y. Wang , Y. Yang , F. Huang , M. Zhu , B. T. W. Ang , J. M. Xue , Adv. Funct. Mater. 2021, 31, 2101607.

[49] R. Zhao , Y. Yang , G. Liu , R. Zhu , J. Huang , Z. Chen , Z. Gao , X. Chen , L. Qie , Adv. Funct. Mater. 2020, 31, 2001867.

[50] Y. An , Y. Tian , S. Xiong , J. Feng , Y. Qian , ACS Nano 2021, 15, 11828. 10.1021/acsnano.1c02928 34133130

[51] L. Suo , D. Oh , Y. Lin , Z. Zhuo , O. Borodin , T. Gao , F. Wang , A. Kushima , Z. Wang , H. C. Kim , Y. Qi , W. Yang , F. Pan , J. Li , K. Xu , C. Wang , J. Am. Chem. Soc. 2017, 139, 18670. 29186955 10.1021/jacs.7b10688

[52] Y. Chu , S. Zhang , S. Wu , Z. Hu , G. Cui , J. Luo , Energy Environ. Sci. 2021, 14, 3609.

[53] R. Yuksel , O. Buyukcakir , W. K. Seong , R. S. Ruoff , Adv. Energy Mater. 2020, 10, 1904215.

[54] A. Naveed , H. Yang , J. Yang , Y. Nuli , J. Wang , Angew. Chem., Int. Ed. 2019, 58, 2760. 10.1002/anie.201813223 30604584

[55] J. Hao , B. Li , X. Li , X. Zeng , S. Zhang , F. Yang , S. Liu , D. Li , C. Wu , Z. Guo , Adv. Mater. 2020, 32, 2003021. 10.1002/adma.202003021 32639067

[56] H. Luo , B. Liu , Z. Yang , Y. Wan , C. Zhong , Electrochem. Energy Rev. 2021, 5, 187.

[57] S. Wu , S. Zhang , Y. Chu , Z. Hu , J. Luo , Adv. Funct. Mater. 2021, 31, 2107397.

[58] S. Zhou , Y. Wang , H. Lu , Y. Zhang , C. Fu , I. Usman , Z. Liu , M. Feng , G. Fang , X. Cao , S. Liang , A. Pan , Adv. Funct. Mater. 2021, 31, 2104361.

[59] S. Guo , L. Qin , T. Zhang , M. Zhou , J. Zhou , G. Fang , S. Liang , Energy Storage Mater. 2021, 34, 545.

[60] M. Liu , L. Yang , H. Liu , A. Amine , Q. Zhao , Y. Song , J. Yang , K. Wang , F. Pan , ACS Appl. Mater. Interfaces 2019, 11, 32046. 31407885 10.1021/acsami.9b11243

[61] H. Yan , S. Li , Y. Nan , S. Yang , B. Li , Adv. Energy Mater. 2021, 11, 2100186.

[62] Y. Jiao , F. Li , X. Jin , Q. Lei , L. Li , L. Wang , T. Ye , E. He , J. Wang , H. Chen , J. Lu , R. Gao , Q. Li , C. Jiang , J. Li , G. He , M. Liao , H. Zhang , I. P. Parkin , H. Peng , Y. Zhang , Adv. Funct. Mater. 2021, 31, 2107652.

[63] B. Lee , H. R. Seo , H. R. Lee , C. S. Yoon , J. H. Kim , K. Y. Chung , B. W. Cho , S. H. Oh , ChemSusChem 2016, 9, 2948. 27650037 10.1002/cssc.201600702

[64] S. Di , X. Nie , G. Ma , W. Yuan , Y. Wang , Y. Liu , S. Shen , N. Zhang , Energy Storage Mater. 2021, 43, 375.

