# Supplementary material for: High‐Rate, Large Capacity, and Long Life Dendrite‐Free Zn Metal Anode Enabled by Trifunctional Electrolyte Additive with a Wide Temperature Range
Source: Adv Sci (Weinh). 2022 May 26;9(21):2201433. doi: 10.1002/advs.202201433 (PMC9313946; doi:10.1002/advs.202201433)
Supplement: Supplementary file 1 — Supporting Information [file ADVS-9-2201433-s002.pdf]

Supporting Information

High-rate, large capacity, and long life dendrite-free Zn metal anode enabled by trifunctional electrolyte additive with a wide temperature range

Chuyuan Lin, Xuhui Yang, Peixun Xiong, Hui Lin, Lingjun He, Qi Yao, Mingdeng Wei, Qingrong Qian, Qinghua Chen* and Lingxing Zeng**

C. Lin, X. Yang, H. Lin, L. He, Q. Yao, Prof. Dr. Q. Qian, Prof. Dr. Q. Chen, Prof. Dr. L. Zeng
Engineering Research Center of Polymer Green Recycling of Ministry of Education, College of Environmental Science and Engineering, Fujian Normal University, Fuzhou, Fujian 350007, People's Republic of China.
E-mail: zenglingxing@fjnu.edu.cn (L. Zeng); cqhuar@126.com (Q. Chen)
Dr. P. Xiong
School of Chemical Engineering, Sungkyunkwan University, Suwon-si, Gyeonggi-do 440-746, Republic of Korea.
E-mail: xiongpeixun@163.com (P. Xiong)
Prof. Dr. Q. Qian, Prof. Dr. Q. Chen, Prof. Dr. L. Zeng
Fujian Key Laboratory of Pollution Control & Resource Reuse, Fuzhou, Fujian 350007, People's Republic of China.
E-mail: zenglingxing@fjnu.edu.cn (L. Zeng); cqhuar@126.com (Q. Chen)
Prof. Dr. Q. Qian, Prof. Dr. Q. Chen, Prof. Dr. L. Zeng
Key Laboratory of Advanced Energy Materials Chemistry (Ministry of Education), College of Chemistry, Nankai University, Tianjin 300071, People's Republic of China.
Prof. Dr. M. Wei
Fujian Provincial Key Laboratory of Electrochemical Energy Storage Materials, Fuzhou University, Fuzhou, Fujian 350002, People's Republic of China.

Keywords: electrolyte additive, homogenize Zn deposition, stable interface pH, Zn anode

1. Experimental

1.1 Characterization

X-ray diffraction (XRD) performed on a Bruker D8 diffractometer by using filtered Cu-Kα radiation under 40 kV and 20 mA. The microstructures of as-prepared samples were investigated by using scanning electron microscopy (SEM) (Hitachi 8010). X-ray photoelectron spectroscopy (XPS) spectra were carried out with an ESCALAB MARK II spherical analyzer with Kα radiation as the X-ray source. Electron paramagnetic resonance measurement was collected on MS 5000. Raman spectra were conducted on DXR2xi with a laser at wavelength of 532 nm.

1.2 Electrochemical Measurements

Tafel plots were scanned within −0.8 and −1.1 V at 1 mV s^{-1} with the reference electrode (Ag/AgCl electrode), and the Zn plates as the counter electrode and the working electrode in three-electrode setups. The chronoamperometry (CA) transient curves were recorded at −150 mV fixed overpotential. Linear sweep voltammetry (LSV) tests were accomplished with a scanning velocity of 10 mV s^{-1}. The above test was implemented in $ZnSO_4$ and $ZnSO_4$: CH_3COONH_4 electrolytes. Electrochemical impedance spectroscopy (EIS) studies were measured at a sinusoidal perturbation of 5 mV and in the frequency range of 1 mHz~100 kHz.

1.3 Computational Methods

Total energy calculations were conducted through density functional theory (DFT) using the Vienna *ab initio* Simulation Package (VASP) using the projector-augmented wave (PAW) method. [S1] The Perdew-Burke-Ernzerhof (PBE)

approximation based on the generalized gradient approximation (GGA) was used to determine the influence of the exchange-correction potentials. [S2, S3] A plane-wave cutoff energy was set as 800 eV. A Monkhorst-Pack K-point sampling of 18×18×6 was used for the crystalline Zn unit cell. To save the computational resources, the Zn (0001) surface was modelled by a five-layer 5×5 surface supercell (25 Zn atoms per layer) considering only considering the Γ point, and the convergence criteria for the ionic relaxation and the electronic self-consistent electronic calculation were set to 0.03 eV/Å and 10^{-5} eV, respectively. In addition, a vacuum of 25 Å was used to avoid the interaction between periodically neighbour slabs. A dispersion-corrected DFT method (DFT-D2) was adopted for the long-range dispersion correction.[S4] For absorption systems, the two top atomic layers were relaxed and the other three bottom layers were fixed.

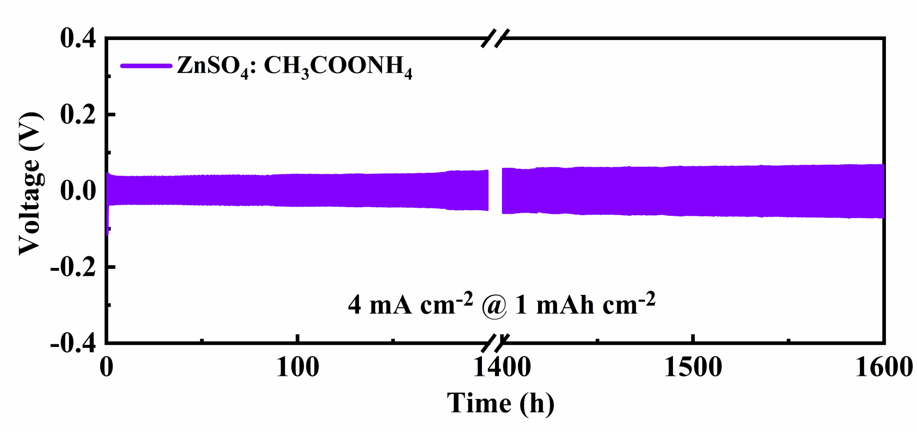

Fig. S1 Cycling performance of Zn||Zn symmetrical cells with $ZnSO_4$: CH_3COONH_4 electrolyte at 4 mA cm^{-2} for 1 mAh cm^{-2}.

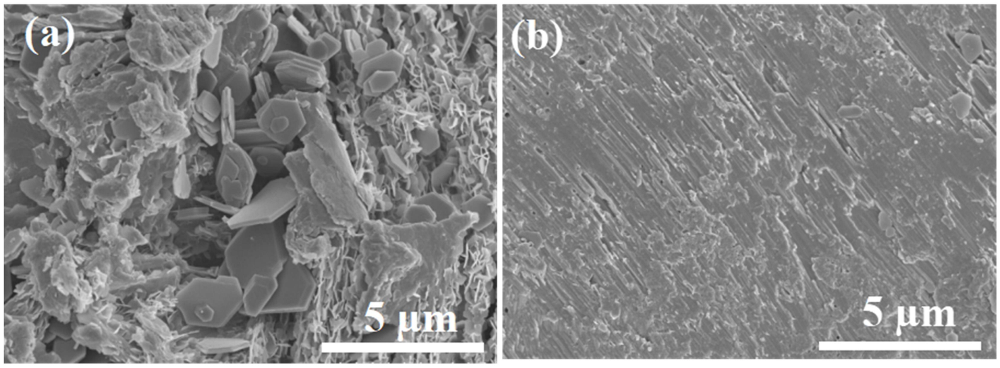

Fig. S2 Scanning electron microscopy (SEM) images of Zn||Zn symmetrical cells cycled 200 h: (a) $ZnSO_4$ electrolyte; (b) $ZnSO_4$: CH_3COONH_4 electrolyte.

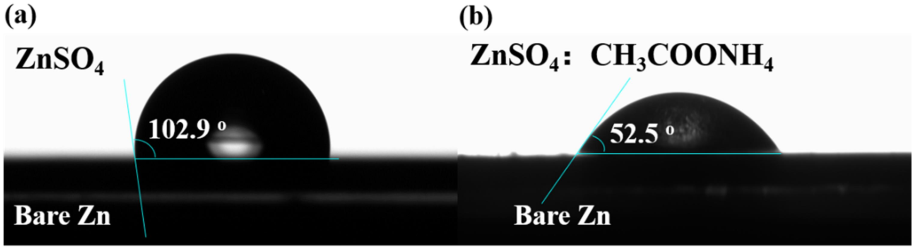

Fig. S3 The contact angle of Zn anode after cycling in (a) $ZnSO_4$ electrolyte and (b) $ZnSO_4$: CH_3COONH_4 electrolyte.

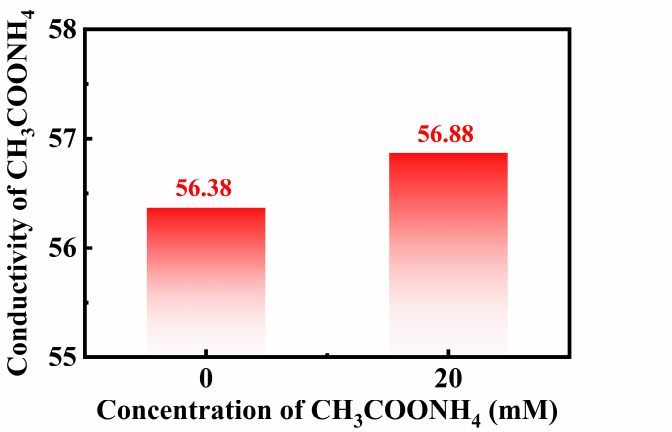

Fig. S4 The conductivity of electrolyte with / without CH_3COONH_4.

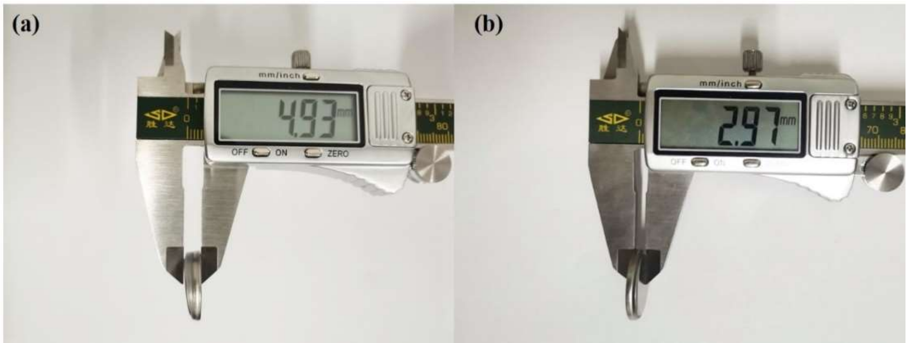

Fig. S5 The photos of the cycled Zn||Zn button cells (a) without or (b) with CH_3COONH_4 after 50 cycles at 20 mA cm^{-2} for 5mAh cm^{-2}.

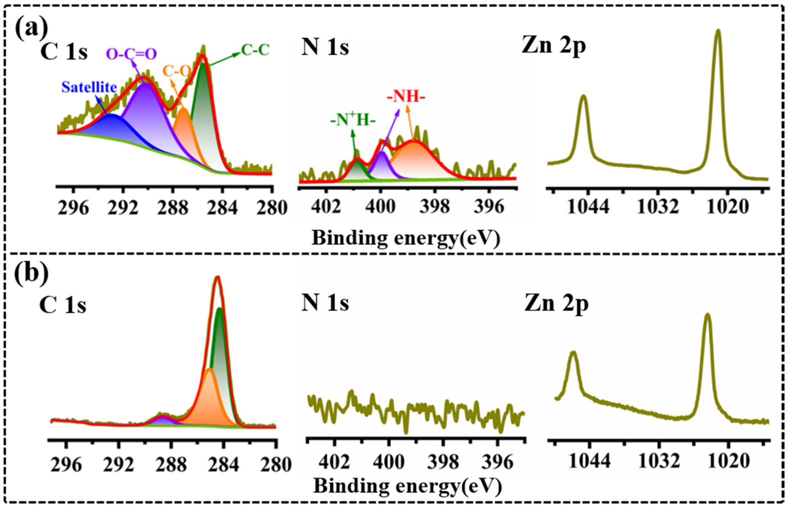

Fig. S6 XPS spectra of C 1s, N 1s and Zn 2p of Zn-electrode after 5 cycles at 1 mA cm^{-2}: (a) $ZnSO_4$: CH_3COONH_4 electrolyte; (b) $ZnSO_4$ electrolyte.

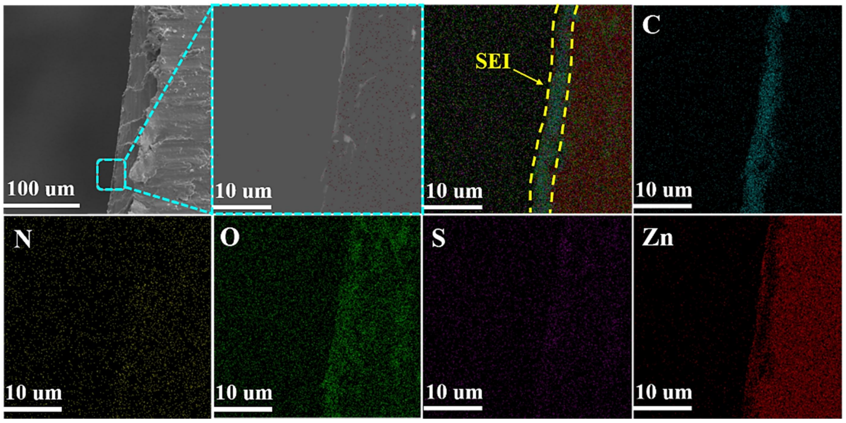

Fig. S7 the scanning electron microscope - energy dispersive spectroscopy (SEM-EDS) images of the cross section of Zn anode after 10 cycles at 1 mA cm^{-2}.

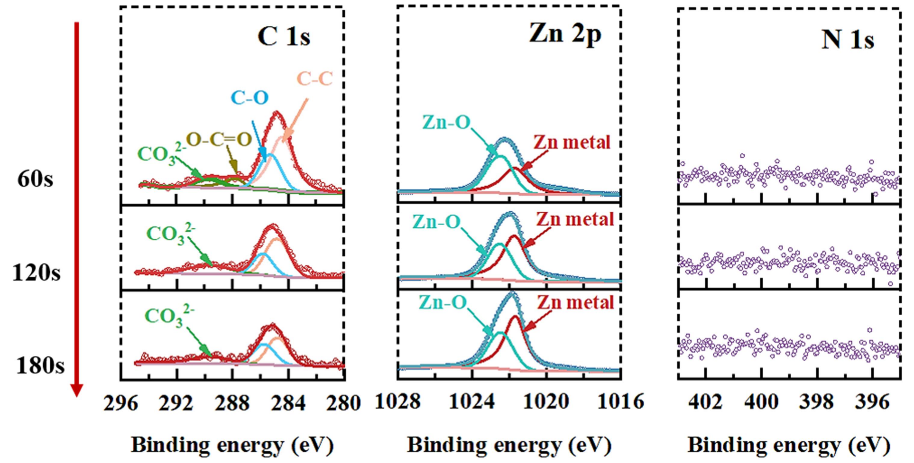

Fig. S8 XPS of C 1s, Zn 2p, and N 1s, which are displayed in rows, with corresponding durations of Ar^+ sputtering in columns.

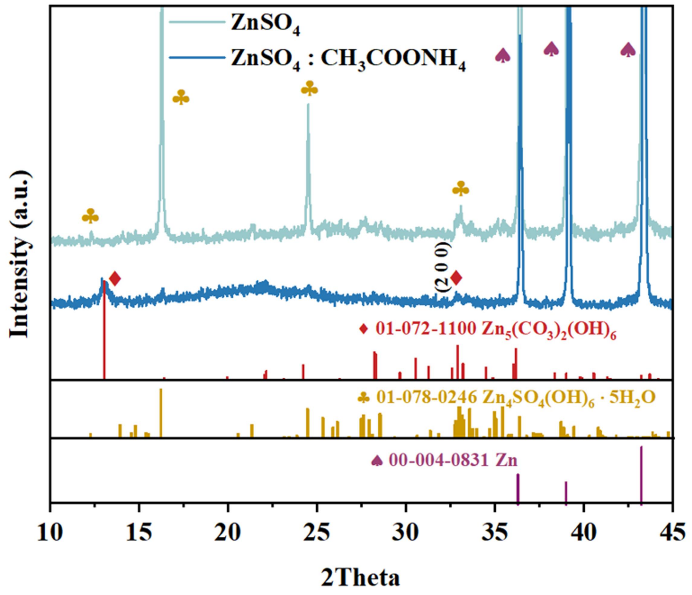

Fig. S9 XRD patterns of Zn anode after 3 cycles in $ZnSO_4$ and $ZnSO_4$: CH_3COONH_4 electrolytes.

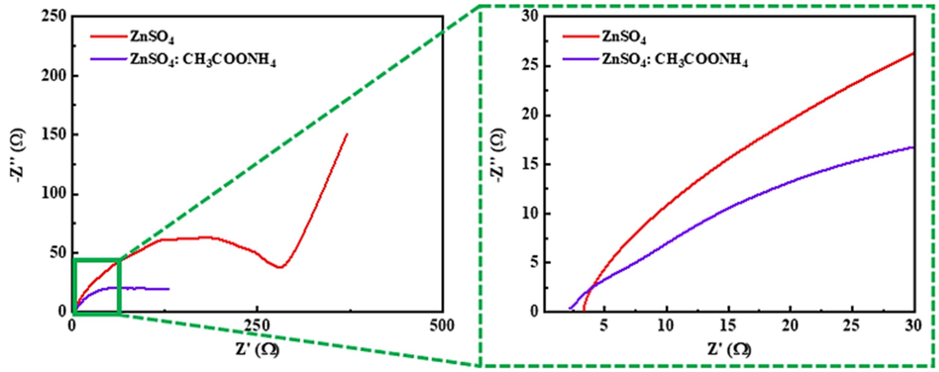

Fig. S10 EIS spectra of the Zn||Zn cells after 5 cycles at 1 mA cm^{-2} for 1 mAh cm^{-2}.

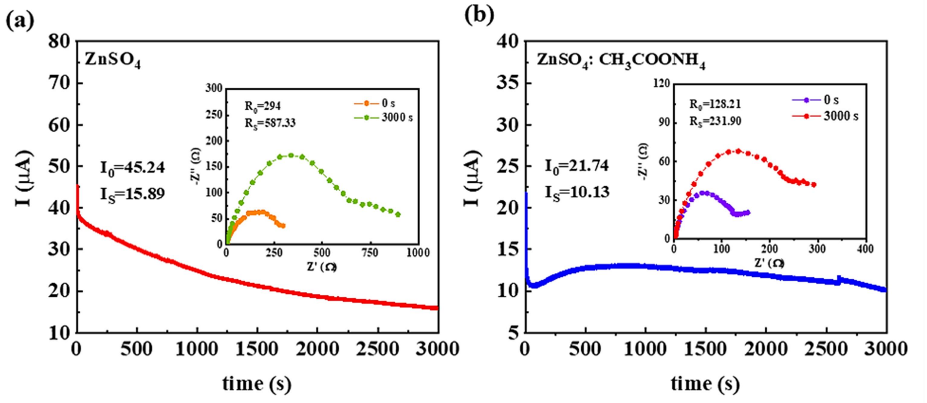

Fig. S11 Measurements of Zn^{2+} transference number. Current-time plots of Zn symmetric cells with (a) bare Zn and (b) SEI-Zn after polarization at 15 mV for 3000 s. The insets are the impedance spectra before and after polarization. The transference number of Zn^{2+} ($t_{Zn^{2+}}$) was evaluated by the following equation[S5]:

$$t_{Zn^{2+}} = \frac{I_S(\Delta V - I_0 R_0)}{I_0(\Delta V - I_S R_S)}$$

where ΔV is the constant polarization voltage applied (15 mV here), I_0 and R_0 are the initial current and resistance, and I_s and R_s are the steady-state current and resistance, respectively.

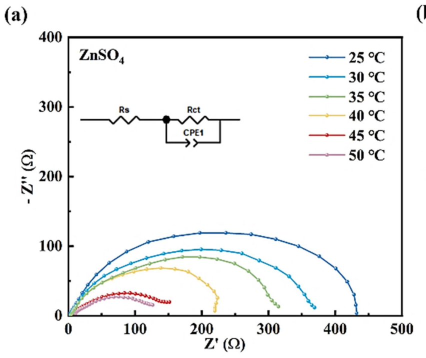

Fig. S12 The corresponding Nyquist plots of Zn symmetric cells in (a) $ZnSO_4$ electrolyte and (b) $ZnSO_4$: CH_3COONH_4 electrolyte at different temperatures.

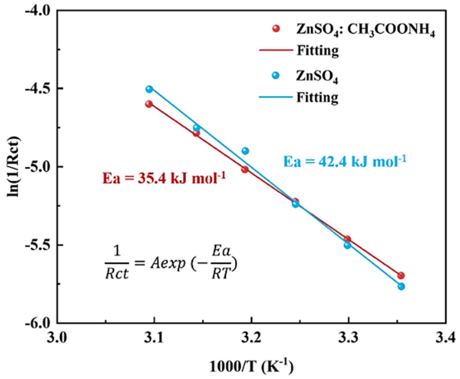

Fig. S13 Arrhenius curves of Zn symmetric cells at different temperatures (inset shows the related equation to calculate the activation energy (Ea)).

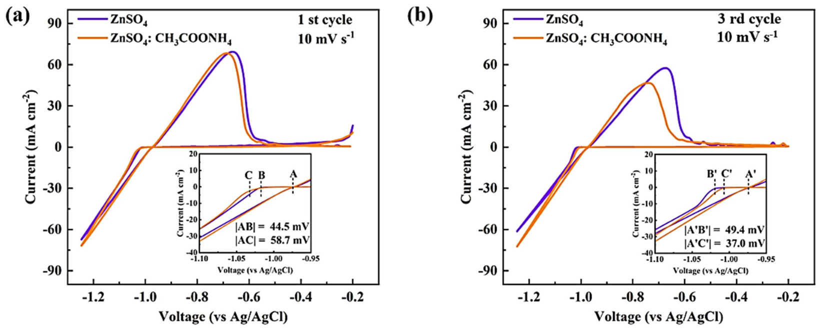

Fig. S14 The cyclic voltammetry (CV) curves of Zn plating/stripping on Ti foil with different electrolytes (10 mV s^{-1}).

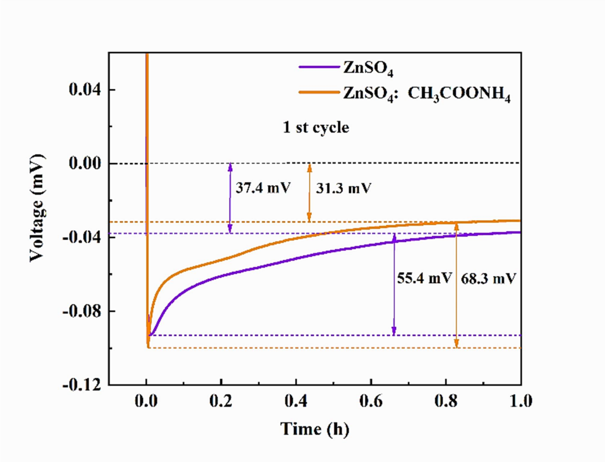

Fig. S15 Nucleation overpotential (NOP) based on the Ti substrate with or without CH_3COONH_4 at a current density of 2 mA cm^{-2}.

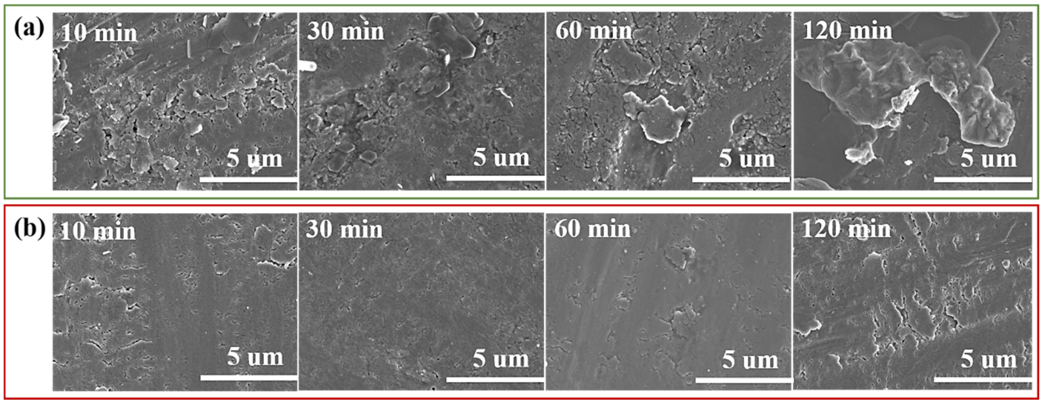

Fig. S16 The SEM image of Ti foil after cycling for 10, 30, 60 and 120 min in (a) $ZnSO_4$ electrolyte and (b) $ZnSO_4$: CH_3COONH_4 electrolyte at 2 mA cm^{-2}.

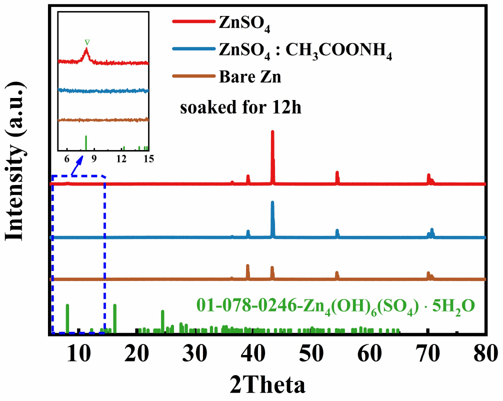

Fig. S17 XRD patterns of pristine Zn foil and Zn-electrode soaked for 12 h in different electrolytes.

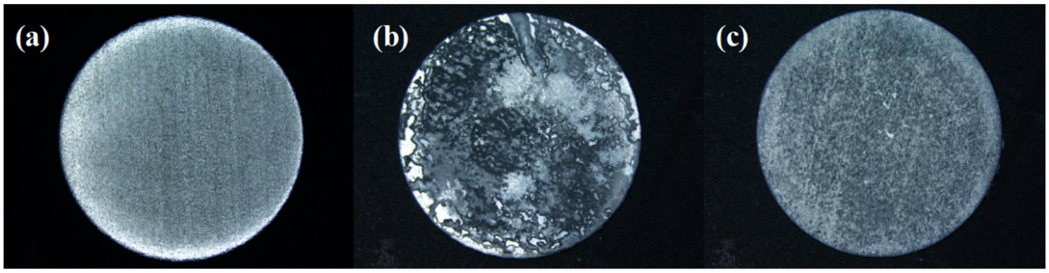

Fig. S18 Photos of (a) pristine Zn foil, (b) Zn-electrode in $ZnSO_4$, and (c) Zn-electrode in $ZnSO_4$: CH_3COONH_4 after 20 cycles at 5 mA cm^{-2} for 5 mAh cm^{-2}.

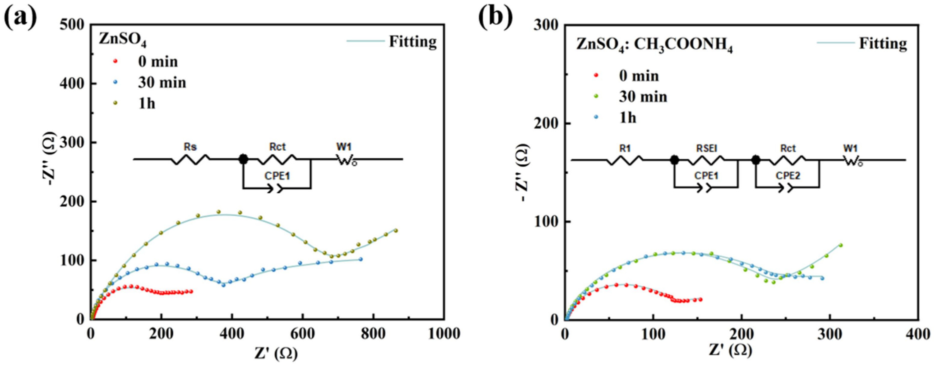

Fig. S19 The EIS fitted curves of the symmetric batteries in difference electrolyte.

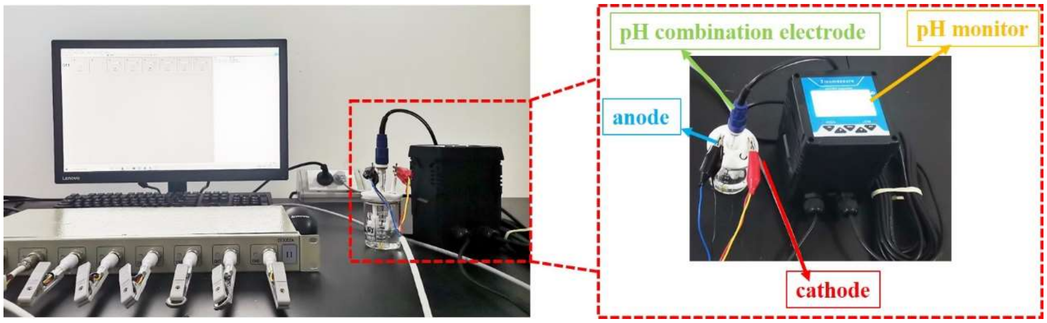

Fig. S20 Digital image of the assembled equipment for studying pH change of electrolytes during cycling.

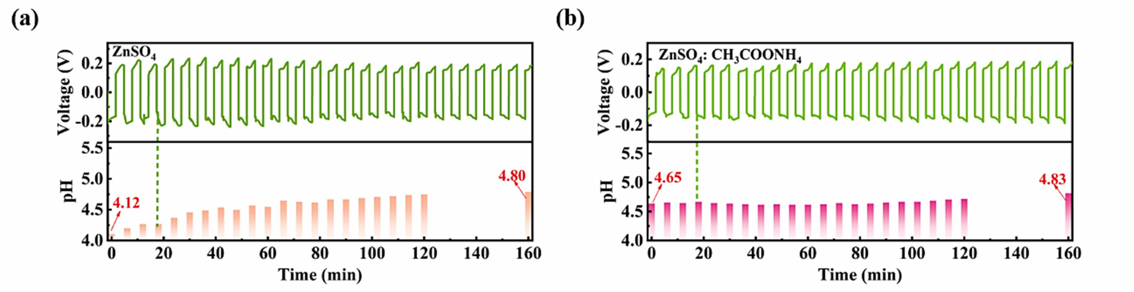

Fig. S21 The pH monitoring of (a) $ZnSO_4$ electrolyte and (b) $ZnSO_4$: CH_3COONH_4 electrolyte of symmetric batteries cycling at 20 mA cm^{-2}, respectively.

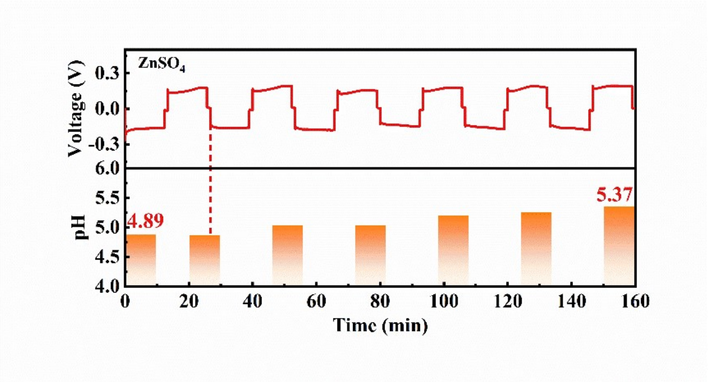

Fig. S22 The pH monitoring of $ZnSO_4$ electrolyte of symmetric batteries cycling at 5 mA cm^{-2} in ice water bath.

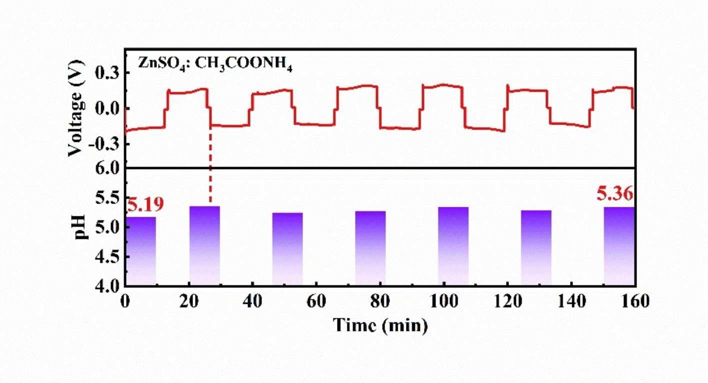

Fig. S23 pH monitoring of $ZnSO_4$: CH_3COONH_4 electrolyte of symmetric batteries cycling at 5 mA cm^{-2} in ice water bath.

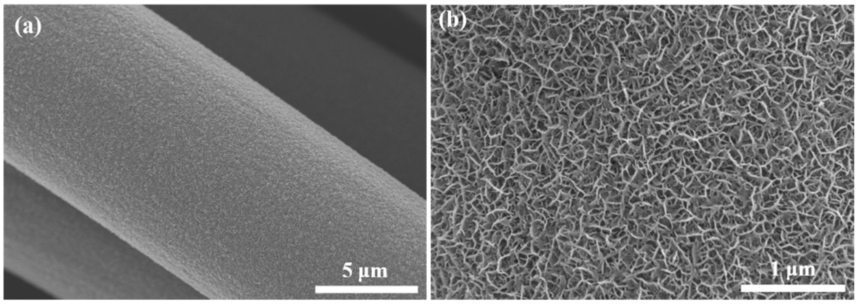

Fig. S24 SEM images of MnO_2 cathode.

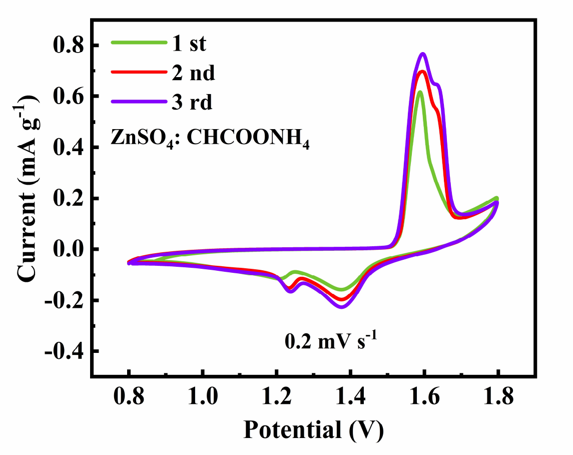

Fig. S25 CV curves of Zn || MnO_2 full cells in $ZnSO_4$: CH_3COONH_4 electrolyte at 0.2 mV s^{-1}.

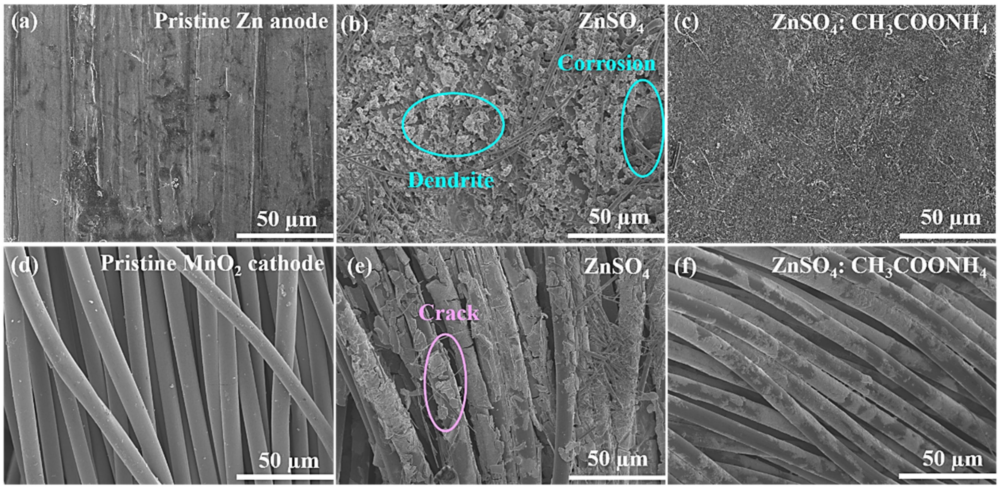

Fig. S26 SEM images of the Zn || MnO_2 full cells after 100 cycles in different electrolyte: (a) Zn anode and (d) MnO_2 cathode before cycling; (b) Zn anode and (e) MnO_2 cathode after cycling in $ZnSO_4$ electrolyte; (c) MnO_2 cathode and (f) Zn anode after cycling in $ZnSO_4$: CH_3COONH_4 electrolyte.

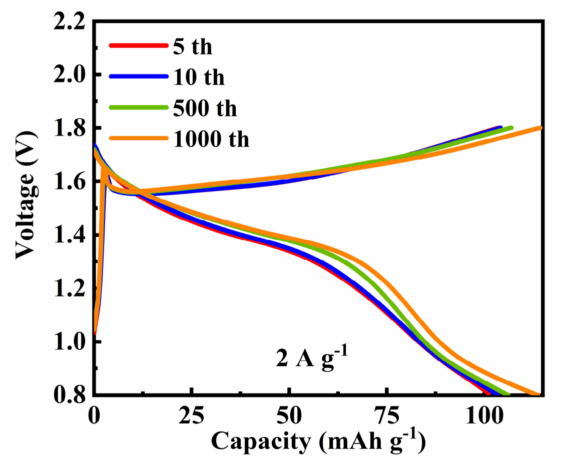

Fig. S27 Charge and discharge curves of Zn || MnO_2 full cells at 2 A g^{-1} for different cycles.

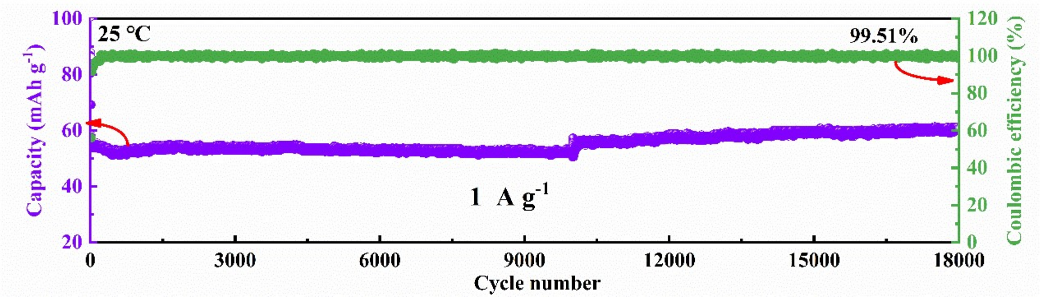

Fig. S28 The coulombic efficiency and long-term cycling performance of Zn||Ac capacitors in $ZnSO_4$: CH_3COONH_4 electrolyte at 25 °C.

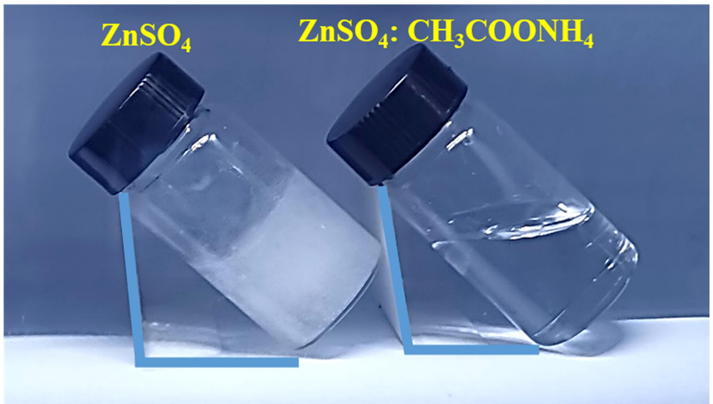

Fig. S29 The digital photos for physical state of $ZnSO_4$ electrolyte and $ZnSO_4$: CH_3COONH_4 electrolytes in -10 °C.

Video S1 Operando compressed fast forward optical video of Zn anodes observed in 2 M $ZnSO_4$ and $ZnSO_4$: CH_3COONH_4 electrolytes for 30 mins.

References

[S1] J. Hafner, *J. Comput. Chem.* **2008**, *29*, 2044.

[S2] G. Kresse, J. Hafner, *Phys. Rev. B* **1993**, *48*, 13115.

[S3] J. P. Perdew, K. Burke, Y. J. P. r. B. Wang, *Phys. Rev. B* **1996**, *54*, 16533.

[S4] S. Grimme, *J. Comput. Chem.* **2006**, *27*, 1787.

[S5] X. Zeng, J. Mao, J. Hao, J. Liu, S. Liu, Z. Wang, Y. Wang, S. Zhang, T. Zheng, J. Liu, P. Rao, Z. Guo, *Adv. Mater.* **2021**, *33*, 2007416.
